# Electronic medical files for patients: some steps towards the future*

**DOI:** 10.1590/S1516-31802010000600001

**Published:** 2010-12-02

**Affiliations:** I Associate professor in the Department of Cardiopneumonology, Faculdade de Medicina da Universidade de São Paulo (FMUSP), São Paulo, Brazil.; II Attending physician in the Thoracic Surgery Service, Instituto do Coração (InCor), Hospital da Clínicas (HC), Faculdade de Medicina da Universidade de São Paulo (FMUSP), São Paulo, Brazil.

There are records of footprints made by hominids in Tanzania 3.6 million years ago. By around 300,000 years ago, the human brain had doubled in size and acquired biological characteristics such as the ability to speak, which placed mankind in a prominent position among terrestrial beings. Speech, along with communication as a whole, has probably been one of mankind's greatest inventions.^1^

Medical files are known by the term *prontuário* in Portuguese, which comes from the Latin *promptuarium*, meaning a place where things that might be needed at any moment are kept. Medical files for possible immediate use are documents as old as the history of medicine itself and the first such documents were produced by the Egyptian physician Inhotep (around 3,000 to 2,500 BC).^2^

Modern man has tools at his disposal for storing and organizing information and making it available. The growth of information and its complexity rapidly lead to a situation of constant challenge in which time is the implacable enemy. At the touch of a few buttons, we can have new information and communication technology at our fingertips, along with electronic support for accessing data on any given subject almost instantaneously. Electronic medical files for patients are no exception and have been shown to be a great challenge in any part of the modern world, forming part of a project that is as necessary as it is intangible, in its broadest conception. While we can imagine the possibility of having all the medical information, or an even more holistic form of information, on any individual's health, at the disposal of physicians and other health promoters at any time and in any place, we can also perceive that such opportunities rarely translate into real options because of the various difficulties relating to their implementation.

An article recently published in the New England Journal of Medicine^3^ classified electronic medical files into three categories or systems: basic, without clinical annotations; basic, with clinical annotations; and advanced, in an integrated manner. According to a questionnaire answered by 3,049 institutions in the United States, the rates of implementation of these systems were 10.9%, 7.6% and 1.5%, respectively. It is of interest to highlight that, in this survey, the great majority of the physicians believed that electronic medical files would be of great value for patient care. It can thus be gathered that although this would be a very welcome tool, there is great difficulty in putting it into practice.

On the other hand, it has already been demonstrated that many of the systems and functional features required for establishing true electronic medical files are already in place and in use in many healthcare units, albeit without any interaction between them.^3^ The greatest difficulties in implementing an integrated system are: lack of capital to acquire such a system; maintenance costs; resistance among physicians; rate of return for a doubtful investment; and insufficiencies in the information technology (IT) team.^3^ Resistance among physicians has been indicated as the greatest barrier among those cited, other than insufficiencies in financial issues, and the main reasons for such resistance relate to losses in productivity and confidentiality of information. Another great difficulty that has been emphasized relates to interaction of the information between different terminals, such as hospitals, clinics, laboratories and consultation offices of physicians and other healthcare professionals.

There are few examples worldwide that could be followed and which would show the feasibility and advantages of adopting such systems. In the United States, the Veterans Health Administration (VHA) network has an implementation rate for an advanced system that is four times greater than the mean rate in that country, and a significant impact on the quality of clinical practice has been noted.^3^ Some countries like the United Kingdom and the Netherlands have high levels of use of health-related information technology, particularly with regard to outpatient attendance.^3^

In Brazil, according to some authors, computerization of medical files has not even reached the stage of being a proposal.^4^ In 2002, the Federal Medical Council (*Conselho Federal de Medicina*, CFM) examined electronic medical files,^5,6^ and made this a regulatory milepost to be followed. Through the CFM's resolutions, it has recognized electronic medical files as a legitimate means of storing patient information. Physicians’ electronic signatures have become recognized on hospital admission, discharge and prescription documents, inserted into electronic medical files.

Medical files are defined as “single documents consisting of information, signs and images recorded from facts, events and situations relating to patients’ health and care provided for such patients, with a legal, confidential and scientific nature, which are used both to enable communication between members of a multiprofessional team and to provide continuity of the care provided to individuals”.^5^ In Brazil, ever since 1873, all medical files have been stored.^5^ This task became the responsibility of the Medical Files and Statistics Service (*Serviço de Arquivo Médico e Estatística*, SAME), and the first unit of this service was established by Hospital das Clínicas, Faculdade de Medicina da Universidade de São Paulo, in 1943.^7^

Furthermore, according to the CFM, paper-based medical files must be kept for a 20-year period after the last records on patients or their death. In addition, there are very specific and stern rules about getting rid of medical files, which make this task unattractive. Thus, it can be imagined what this means in quantitative and qualitative terms regarding the storage of so much information. Electronic medical files are a good alternative for a large proportion of these problems. According to a survey published in 2003,^4^ the Brazilian scenario does not differ from the picture in other countries: computerization of administrative routines, in activities such as making appointments for consultations, patient registration and reimbursement of expenses, is already a reality. However, replacement of paper-based medical files has not yet become an attainable proposal at national level.^4^ Some Brazilian hospitals have shown concern regarding this issue and have achieved solid advances. The Cancer Institute of the State of São Paulo (*Instituto do Câncer do Estado de São Paulo*, Icesp) has been one of the pioneers in implementing electronic medical files.^8^ The paper model was replaced by a computerized system that was acquired in August 2008. To sign the electronic medical files, physicians need to have digital certification, which represents their signature and identity in the virtual world and is equivalent to their traditional registration number with the Regional Medical Council (*Conselho Regional de Medicina*, CRM). The executive director of Icesp, Marcos Fumio, explains that this project was conceived soon after the Institute began its activities. From this experience, they have acquired much knowledge regarding the advantages of electronic medical files, such as the legibility of information; access by different professionals at times and locations that may or may not be different; sustainability and environmental issues; and, especially, confidentiality and reliability. According to the information technology director of Icesp, Kaio Bin, “All the information entered by physicians into the medical file generate an encrypted code that is linked to their login in the system and is stored on a lead plate”. Thus, the digital signature proves who filled out the medical files and when this was done, which is impossible with conventional medical files. He explains that the digital signature is safer, “because it is easier to adulterate a rubber stamp than an encrypted code”. Marcos Fumio also notes that the system implemented at Icesp may become even more productive through the possible implementation of the National Health System's SUS card (*Sistema Único de Saúde*). “If the way in which patient data is stored were to be standardized, the information could be consulted at any hospital”.

Some institutions serve as examples of partial implementation, with major advances but still dependent on paper-based medical files. Thus, Instituto do Coração (InCor), which is part of Hospital das Clínicas, Faculdade de Medicina da Universidade de São Paulo, has advanced significantly towards integrating a hospital information system that stores administrative and clinical information with a system for transmission, storage, retrieval, processing and viewing medical images. From the experience acquired by InCor,^9^ it has been concluded that the big challenge in fully adopting electronic medical files, going through physicians’ scrutiny, is the credibility of the integrated systems. Credibility implies concern regarding the ethical issues of secrecy and confidentiality, along with the legal violation that is intrinsic to medical files.

Confidentiality of the information held in medical files is every citizen's right, with backing from the Federal Constitution of 1988, in article 5, item X, which guarantees that individuals’ intimacy, private lives, image and honor are inviolable.^10^ This duty to preserve confidentiality is foreseen in the Brazilian Penal Code, article 154, and in most healthcare professionals’ ethical codes.^11^ The Medical Ethics Code, article 11,^12^ lays down that confidentiality is a fundamental principle for practicing medicine. Chapter IX of this code covers the obligations regarding professional confidentiality.^13^ Among these obligations are physicians’ duty to guide their assistants and be watchful to ensure that everyone respects professional confidentiality, along with prohibiting physicians from facilitating access to medical files by persons who do not have an obligation to preserve professional confidentiality. In principle, only patient consent can authorize disclosure of medical file contents, through the principle of autonomy.^5^ Patients would decide which information to keep to themselves and which information they wished to reveal. However, in the Medical Ethics Code itself, article 102, there is a proviso regarding such disclosure, such that it can be done “on justifiable grounds, as a legal duty, or through express authorization by the patient”. Not only physicians but also nurses and other healthcare professionals, along with all administrative employees who come into contact with patient information as part of their duty, are authorized to access this information only insofar as there is a professional need to do so. Therefore, the duty to ensure confidentiality is not limited to physicians, but extends to all workers who, because of their profession, may have access to these data.

Although doubts still persist regarding electronic signatures, digital certification and other questions of “credibility”, all the indications are that the good experiences obtained have promoted one of the greatest revolutions relating to patient care, along with making it possible to use the information for teaching and scientific purposes. We believe that the first steps towards the future of electronic medical files for patients have been very promising.
